# The Anthelmintic Ingredient Moxidectin Negatively Affects Seed Germination of Three Temperate Grassland Species

**DOI:** 10.1371/journal.pone.0166366

**Published:** 2016-11-15

**Authors:** Carsten Eichberg, Manuel Wohde, Kerstin Müller, Anja Rausch, Christina Scherrmann, Theresa Scheuren, Rolf-Alexander Düring, Tobias W. Donath

**Affiliations:** 1 Regional and Environmental Sciences, Geobotany, University of Trier, Trier, Germany; 2 Institute of Soil Science and Soil Conservation, Justus Liebig University, Gießen, Germany; 3 Department of Landscape Ecology, Institute for Natural Resource Conservation, Christian Albrechts University Kiel, Kiel, Germany; Banaras Hindu University, INDIA

## Abstract

In animal farming, anthelmintics are regularly applied to control gastrointestinal nematodes. There is plenty of evidence that also non-target organisms, such as dung beetles, are negatively affected by residues of anthelmintics in faeces of domestic ungulates. By contrast, knowledge about possible effects on wild plants is scarce. To bridge this gap of knowledge, we tested for effects of the common anthelmintic formulation Cydectin and its active ingredient moxidectin on seed germination. We conducted a feeding experiment with sheep and germination experiments in a climate chamber. Three wide-spread plant species of temperate grasslands (*Centaurea jacea*, *Galium verum*, *Plantago lanceolata*) were studied. We found significant influences of both, Cydectin and moxidectin, on germination of the tested species. Across species, both formulation and active ingredient solely led to a decrease in germination percentage and synchrony of germination and an increase in mean germination time with the formulation showing a more pronounced response pattern. Our study shows for the first time that anthelmintics have the potential to negatively affect plant regeneration. This has practical implications for nature conservation since our results suggest that treatments of livestock with anthelmintics should be carefully timed to not impede endozoochorous seed exchange between plant populations.

## Introduction

Gastrointestinal nematodes are a major cause of ill health and poor productivity in domestic ungulates, such as sheep and cattle [1]. In order to control nematodes, a broad spectrum of anthelmintics is administered to domestic ungulate species worldwide. Among anthelmintics, macrocyclic lactones currently play a central role in controlling parasites because they have a broad action spectrum against endo- and ectoparasites, can be administered to various livestock species (e.g. sheep, cattle, horse) and have little effect on mammals [2]. Worm resistance to these drugs is relatively low [3–6]. Macrocyclic lactones are brought into the environment mainly via faeces excretion [7]. They are excreted mainly as parent (unchanged) drug and adversely affect many non-target organisms, especially arthropods [8–9]. The strength of the effect depends on the active ingredient, its formulation and concentration. Phytotoxicity of anthelmintics has been investigated very rarely (reviewed in [4, 10]). The effect of anthelmintics (moxidectin) on seed germination was tested only by one authorisation study which used specific laboratory conditions and did not include the gastrointestinal passage and faeces substrate [11].

In grazed open vegetation, the seeds of many plant species are dispersed endozoochorously by ungulates, some in high densities [12–16]. Like other seed dispersal mechanisms, endozoochory is a multi-step process; it includes diet selection, mastication, seed passage through digestive tract, spreading of seeds, stay of seeds in above-ground faecal deposits or burial by dung beetles, germination and seedling establishment. Although endozoochorous dispersal is a costly dispersal mechanism with high losses of individual numbers in many phases [17–19], it is regarded as important for the establishment and maintenance of plant populations in grazed ecosystems [20]. Especially in todays fragmented cultural landscapes dispersal limitation is a major hindrance for the restoration of species-rich grasslands [21]. In this context, nature conservation tries to take advantage of the high capability of sheep to transport seeds, both internally and externally (fur, hooves), from one place to another. Roaming livestock, especially sheep, are proven to be effective seed dispersal vectors (sensu [22]), i.e. their dispersal activity leads to a successful establishment of plant individuals at new sites (reviewed in [20]). Therefore, any direct or indirect negative effects on the effectiveness of seed dispersal by livestock will be of relevance for nature conservation.

Negative effects of macrocyclic lactones on decomposers are well studied [8–9, 23–24] and from these findings restrictions in nutrient circulation and pasture quality can be deduced [4]. A reduction in faeces breakdown also has consequences for the success of endozoochorous seed dispersal since physical fragmentation of faeces, e.g. by ungulate trampling, has been shown to facilitate seedling emergence and establishment of faeces-embedded seeds [25–26]. Besides indirect effects of anthelmintics on seedling emergence, direct (i.e. toxic) effects might exist. In a previous study on the post-dispersal establishment success of two *Plantago* species (*P*. *lanceolata*, *P*. *major*; Scherrmann & Eichberg, unpublished data), including a factor combination of faeces and trampling, we observed surprisingly low numbers of seedlings emerging out of sheep faeces. Getting back to the shepherd she recounted that the sheep had been treated with anthelmintics (Cydectin^®^ 0.1% oral drench for sheep, Zoetis Deutschland GmbH, Berlin) two weeks before the analysed faeces were sampled. Therefore, we tested the assumption that the anthelmintic might have caused this result. This assumption has so far generally not been tested under realistic conditions.

To close this gap of knowledge, we conducted three experiments focusing on the effects of the anthelmintic Cydectin and its active pharmaceutical ingredient moxidectin, a common macrocyclic lactone used in livestock farming [27], on seed germination. First, we conducted a feeding experiment with the typical grassland species *Plantago lanceolata* where we tested the effects of anthelmintics on the number of seedlings emerging out of faeces from sheep treated with Cydectin. Second, we exposed seeds of three common grassland species (*Centaurea jacea*, *Galium verum*, *P*. *lanceolata*) to different concentrations of Cydectin as well as moxidectin in two germination experiments and assessed germination percentage, mean germination time and synchrony of germination [28–29]. Within this paper we define ‘germination’ as the appearance of the radicle (germination experiments) or cotyledons (feeding experiment), although there is a short time difference between these early stages of seedling development. We use a broad definition because we were generally interested in effects of anthelmintics on seeds without aiming at distinguishing these stages.

We addressed the following questions:

- Do anthelmintics adversely affect germination of temperate grassland species?
- If an effect is given, are there differences in the effect of the active pharmaceutical ingredient and the formulation?

## Materials and Methods

### Test substance

We tested the anthelmintic formulation Cydectin^®^ 0.1% oral drench for sheep (Zoetis Deutschland GmbH, Berlin) and its active pharmaceutical ingredient moxidectin, a chemically optimised fermentation product of the soil bacterium *Streptomyces cyaneogriseus* subsp. *noncyanogenus* [30]. Moxidectin is applied worldwide to domestic ungulate species against endo- and ectoparasites [2]. In contrast to other macrocyclic lactones (such as ivermectin), moxidectin has a long efficacy time in the sheep body [31]. It accumulates in the fat tissue and has a residue depletion half-life of 13.5–15.0 d after oral administration [32]. The major elimination pathway is via faeces whilst elimination via urine is negligible [32]. Macrocyclic lactones persist in faeces environment for a long time at a concentration high enough to affect health of non-target organisms [4]; however, moxidectin in particular, has a comparatively low toxicity against non-target insects [24]. Macrocyclic lactones bind to glutamate-gated chloride channels and enhance permeability for chloride ions through membranes of nerve and muscle cells of invertebrates [5]. The influx of chloride ions leads to paralysis and death of the parasite [30]. However, the mode of action of macrocyclic lactones is still not completely understood [2, 4].

### Seed material

We used seeds of three herbaceous, perennial vascular plant species typical for Central European grasslands: *Centaurea jacea* L. (Asteraceae), *Galium verum* L. (Rubiaceae), *Plantago lanceolata* L. (Plantaginaceae). Natural endozoochorous dispersal by sheep has been shown to occur for these species [13]. Seeds were obtained from a commercial supplier (Appels Wilde Samen GmbH, Darmstadt, Germany). In case of *P*. *lanceolata*, seeds from the same lot have been used for both experiments.

### Feeding experiment

In a feeding experiment, we aimed at testing for anthelmintic effects *in vivo* on post-digestion seed germination. To this end, sheep were fed with a defined quantity of *P*. *lanceolata* seeds and treated with Cydectin by the shepherd (assisted by T. Scheuren). *Plantatgo lanceolata* is preferably eaten by sheep and seeds of this species are part of the sheep’s regular diet [13, 33]. The anthelmintic tested was administered to the sheep in accordance to the regular treatment cycles and dosed as recommended by the producer. Therefore, in accordance to the German Animal Welfare Legislation Act (TierSchG, TierSchVersV), the present feeding experiment did not need approval by an ethical committee or by the government since this type of investigations did not induce pain, suffering or damages to the animals.

On April 25, 2014, 12 adult female sheep of similar age (ca. 1.5 yr) and body weight (ca. 60–70 kg) were each fed with defined portions of seeds of *P*. *lanceolata* (6.8 g corresponding to 4,510 seeds; thousand seed weight: 1.51 g (mean of three replicates)). Directly afterwards, seven of these sheep were treated with Cydectin 0.1% following the recommendations of the producer (0.2 mg moxidectin kg^-1^ body weight), whereas five sheep remained untreated (control group). The sheep were randomly assigned to one of these two groups. We applied the seed portions (together with tap water) onto the back of the throat by an enema over the tongue. The same technique has been used to administer the Cydectin solutions for the treated group of sheep. Although this technique has the disadvantage of skipping the mastication process during feeding, in ruminants swallowed seeds get treated by teeth while ruminating, which lasts 5–9.5 h per day in sheep [34].

In order to prevent contamination by seeds of wild plant individuals of *P*. *lanceolata*, we conducted our experiment prior to fruiting of the study species (flowering period in Germany: May-Sept; [35]). In addition, we gained four faeces samples from the sheep immediately before experimental seed application and tested them for seed content under the same conditions as the post-treatment faeces samples. No *P*. *lanceolata* seedlings emerged (appearance of cotyledons) out of these samples.

After dosing, the two sheep groups were kept on separate paddocks bearing the same type of mesic grassland (location: Hadamar-Niederzeuzheim, Hesse, Germany, 50°28'4.90"N, 8°1'55.53"E). Over a 1-wk period (26.4.-2.5.14) faeces samples were collected daily from the ground of the two paddocks. The period length was generously adjusted to seed retention time in sheep: In previous feeding experiments, most seeds were excreted by sheep after 2–3 days [17, 36]. Faecal pats had different numbers of pellets and therefore sample sizes varied. To guarantee sufficient amounts of pellets per sample, we collected all pellets that were found within a 1-m radius as a bulk sample at spots where fresh faeces occurred. All detectable fresh faeces heaps were sampled per day and paddock. Fresh faeces pellets were easily distinguishable from old pellets by their shiny surface. For the reason of animal welfare, the sheep of the control group were treated with Cydectin in the same manner as the sheep of the treated group immediately after the last faeces were collected. There were no adverse outcomes observed in the animals from the one-week delay of receiving Cydectin.

All faeces samples were stored at a temperature of 5°C for 4–5 d. Although, we did not observe any seeds sticking to the pellets, faeces samples were washed gently with a water jet to ensure that no *P*. *lanceolata* seeds of the seed or litter bank of previous years stuck to the pellets’ surface.

Per faeces collection, a subsample of 20 g has been taken aside to assess water content (drying at 105°C for 28 h). The remaining material of each sample (30 ± 15 g dry weight, mean ± SD; n = 93) was crushed gently and spread out over a 2-cm layer of steam-sterilised soil (pH 0.01 M CaCl_2_: 4.7) from a common pasture with occurrence of *P*. *lanceolata* in perforated plastic trays (18.5 cm x 28.5 cm). Beforehand, the bottom of the trays was covered with a layer of fleece to prevent seed loss. Trays were randomly positioned on a table in a greenhouse and their position was changed randomly from time to time. Trays were watered from above and below as required (usually three times per week). Seedlings that emerged were counted and removed daily over an 18-wk period (May-Sept. 2014; all samples had the same exposition duration). During exposition period, average air temperature was 20 ± 5°C (mean ± SD of the measurements of two HOBOPro v2 data logger; Onset Computer Corporation, Massachusetts, USA; Min. 8°C, Max. 38°C). Seventeen control trays with steam-sterilised soil only were placed regularly between sample trays; in these trays no *P*. *lanceolata* seedlings emerged.

### Germination experiments

Two parallel germination experiments were carried out in a climate chamber. In a first germination experiment, moxidectin was applied as a pure substance. Moxidectin has a low solubility and stability in water and shows a high tendency to adsorb to surfaces out of aqueous solutions [11]. Therefore, stock solutions were prepared in ethanol (5, 50, 500, 5000 mg l^-1^). Each treatment solution was generated by diluting 10 μl stock solution with 5 ml distilled water, resulting in moxidectin concentrations of 0.01, 0.1, 1 and 10 mg l^-1^ (moxidectin: CAS: 113507-06-5, Sigma-Aldrich, purity: 96.4%; ethanol: Carl Roth, purity: 99.8%). A solvent control was prepared (10 μl ethanol + 5 ml distilled water). In a second germination experiment, moxidectin was given to the treatment solutions in its formulated form Cydectin (0.1% moxidectin). Cydectin was diluted with water to concentrations of 1 and 10 mg l^-1^. Additionally, a blank control was prepared (5 ml distilled water).

In both experiments, each treatment was replicated ten times. In each species, 50 seeds were spread on filter paper in a glass Petri dish (9 cm diameter) to minimise adsorption and 5 ml of the respective treatment solution was applied. Dishes were exposed in a climate chamber at a 15/5°C (16/8 h) day/night regime over a 5-wk period. Emerging seedlings (appearance of radicle) were counted in regular intervals twice a week.

To our knowledge there are no values available on the concentrations of moxidectin in the gastrointestinal tract of sheep or in sheep faeces (locations of seed exposition to anthelmintics) in literature. Afzal et al. [32] treated sheep orally with 0.2 mg moxidectin kg^-1^ body weight and found an active ingredient concentration of 0.005 mg l^-1^ in blood and up to 0.277 mg l^-1^ in fat tissue on day-1 post treatment. Lloberas et al. [27] used the same treatment dose but the intraruminal administration route and measured mean moxidectin concentrations of 0.231 mg l^-1^ (0.5 days post treatment) in the intestinal mucosa of lambs. In faecal pats of cattle (treatment dose: 0.2 mg moxidectin kg^-1^ body weight, subcutaneous administration) mean concentrations of 0.645 mg l^-1^ were found on day-3 post administration [9]. The range of concentrations used in our study encompasses these values.

### Statistical analyses

For the data analyses of the feeding experiment we employed a two-way analysis of variance (ANOVA) with *anthelmintic* (*k* = 2; anthelmintic vs. no anthelmintic) and *time*, i.e. day of faeces collection (*k* = 2, day-1 and -2 post treatment) as predictor variables and *number of seeds germinated* as the response variable. Since 97% of the seedlings emerged from faeces collected during the first two days after anthelmintic application, only these data were included in the analyses, i.e. 16 faeces samples of treated sheep and 12 faeces samples of untreated sheep.

In case of the germination experiments, we calculated *germination percentage* (GP; %), *mean germination time* (MGT; days) and *synchrony of germination* (Z; dimensionless) per replicate [28–29] as response variables. GP is calculated as the percentage of germinated seeds from the initial number of seeds. Calculations of MGT and Z were based on seedling counts over time [29]. Mean germination time is a measure of the weighted average length of time required for germination [28]. The unit depends on the counting frequency, which is days in the present study. Synchrony of germination indicates the germination variability during the experiment and ranges from 0 to 1. The higher the values, the more synchronous the germination is. We analysed the effects of the predictor variables *species* (*k* = 3; *C*. *jacea*, *G*. *verum*, *P*. *lanceolata*) and *treatment* (Cydectin: *k* = 3; moxidectin: *k* = 5) on GP, MGT and Z with a two-way analysis of variance (ANOVA). As the solvent control for the analyses we used the 5 ml water + 10 μl ethanol treatment since we were interested in the effect of the anthelmintic and not the combined effect of anthelmintic and ethanol on the germination variables. Statistical analysis revealed no difference in germination percentage between the two treatments without addition of anthelmintics (5 ml water only vs. 5 ml water + 10 μl ethanol; ANOVA across species, *F*_1,59_ = 0.75, *P* = 0.39).

In order to assess the effect of the anthelmintic on the germination variables, i.e. GP, MGT and Z, we calculated contrasts between the anthelmintic treatments and the control treatment. Contrast analyses were also applied to test for differences in the effect of the active ingredient at moxidectin concentrations of 1 mg l^-1^ and 10 mg l^-1^ when applied solely or formulated in Cydectin.

Prior to analyses, data were transformed to improve normal distribution and homogeneity of variance [*seedling number*: Box-Cox-transformation; *germination percentage*: arcsin (square root/100); *mean germination time* and *synchrony*: Box-Cox-transformation]. All statistical tests were conducted using STATISTICA 12 (StatSoft Inc., Tulsa, Oklahama, USA).

## Results

### Feeding experiment

**Fig 1:**
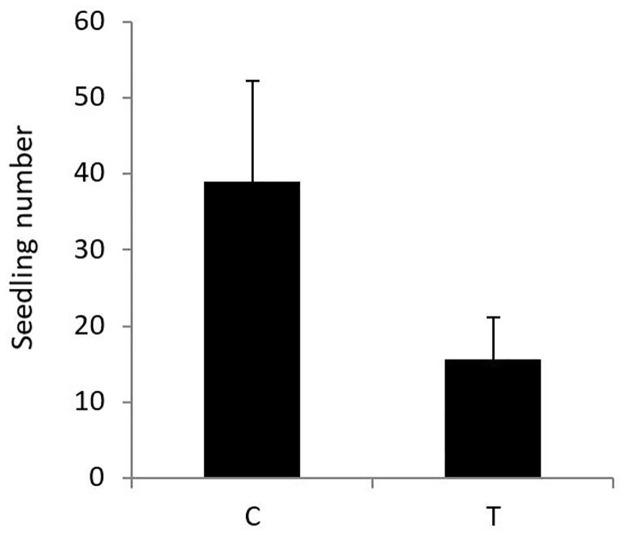
Effects (mean + SE) of treatments (C = untreated control, T = treated with Cydectin) on number of seedlings of *Plantago lanceolata* emerging out of sheep faeces (seedlings 100 g^-1^ dry faeces). Difference between C and T is significant at *P* ≤ 0.05 (cf. Table 1).

The number of seedlings emerging out of faeces of sheep treated with Cydectin was significantly lowered by almost two thirds compared to seedling emergence out of faeces of untreated sheep (Table 1, Fig 1). Additionally, day of faeces collection had influence on seedling number, but did not interact with anthelmintic application.

**Table 1:** Results of the ANOVA for the feeding experiment with faeces collected from sheep that were fed with *Plantago lanceolata* seeds.

	Seedling number			
	df	MS	*F*	*P*
Intercept	1	88.1	89.27	< 0.0001
Treatment [T]	1	4.2	4.30	0.049
Day [D]	1	33.4	33.83	< 0.0001
T × D	1	0.02	0.02	0.88
Residuals	24	0.99		

Effects of treatment (Cydectin applied vs. no Cydectin applied), day of faeces collection and the interaction of these factors on seedling number are shown. df = degrees of freedom, MS = mean square, *F* = variance ratio, *P* = error probability.

### Germination experiments

#### Cydectin

**Fig 2:**
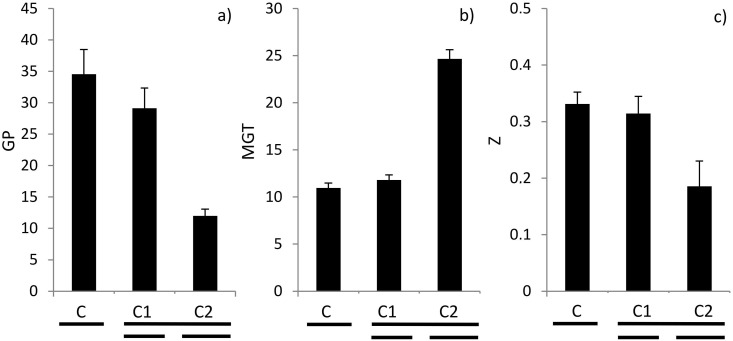
Effects (mean + SE) of treatments (C = control, C1 = 1 mg l^-1^ moxidectin formulated in Cydectin, C2 = 10 mg l^-1^ moxidectin formulated in Cydectin) on a) germination percentage (GP) [%], b) mean germination time (MGT) [days] and c) synchrony of germination (Z) [unitless] across species. Lines below the bars indicate significance as revealed by contrast analyses; broken lines indicate significant differences at *P* ≤ 0.05.; the upper lines indicate significance between C vs. C1 and C2, and the lower lines indicate differences between C1 and C2.

All three response variables (GP, MGT, Z) were significantly influenced by the Cydectin treatment (Table 2). The analyses also revealed a significant species × treatment interaction for GP and MGT. However, when looking at each species separately, it seems that their response towards Cydectin was similar (S1 Fig). Therefore, this is a quantitative rather than a qualitative interaction that only indicates differences in strength but not direction of the effect, i.e. in all species GP decreased, MGT increased and Z decreased (exception in Z: *G*. *verum*) at higher Cydectin concentrations. At a Cydectin concentration that equals 10 mg l^-1^ moxidectin, germination was reduced by two thirds compared to the control treatment (Fig 2). The same treatment more than doubled MGT and reduced Z by one third compared to the control. Although both contrasts were significant, the response patterns suggested that mainly the highest Cydectin concentration was responsible for this result (Fig 2).

**Table 2:** Results of the ANOVA for the germination experiment with two different concentrations of Cydectin (1 mg l^-1^/10 mg l^-1^).

		GP			MGT			Z		
	df	MS	*F*	*P*	MS	*F*	*P*	MS	*F*	*P*
Intercept	1	20.32	1766.3	< 0.0001	26859.0	2060.4	< 0.0001	3.31	384.8	< 0.0001
Species [S]	2	0.74	64.22	< 0.0001	160.4	12.31	< 0.0001	0.02	1.74	0.18
Treatment [T]	2	0.58	47.60	< 0.0001	2149.5	164.89	< 0.0001	0.07	8.68	< 0.0001
S × T	4	0.13	11.10	< 0.0001	69.7	5.34	0.0008	0.02	2.24	0.07
Residuals	76	0.01			13.0			0.01		

Effects of species, treatment and the interaction of these factors on germination percentage (GP), mean germination time (MGT) and synchrony of germination (Z) are shown. Further abbreviations see Table 1.

#### Moxidectin

**Fig 3:**
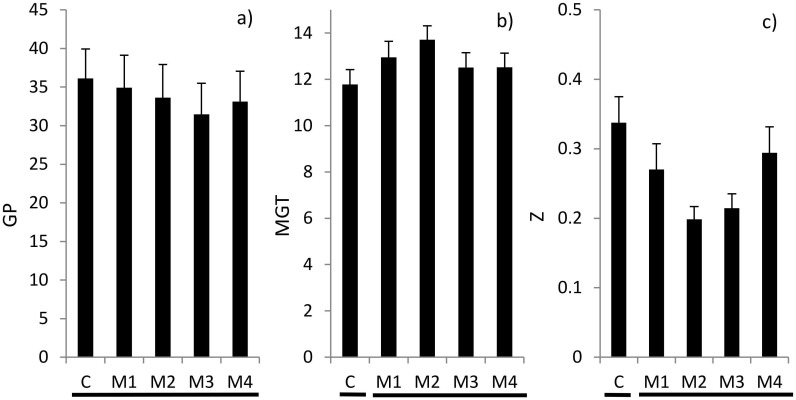
Effects (mean + SE) of treatments (C = control, M1 = 0.01 mg l^-1^ moxidectin, M2 = 0.1 mg l^-1^ moxidectin, M3 = 1 mg l^-1^ moxidectin, M4 = 10 mg l^-1^ moxidectin) on a) germination percentage (GP) [%], b) mean germination time (MGT) [days] and c) synchrony of germination Z [unitless] across species. Lines below the bars indicate significance as revealed by contrasts of C vs. M1, M2, M3 and M4; broken lines indicate significant differences at *P* ≤ 0.05. No significant differences (*P* ≤ 0.05) were detected among treatments M1 to M4.

Across species, the calculated germination variables responded to increasing moxidectin concentrations, i.e., as in case of the anthelmintic formulation, GP decreased, MGT increased and Z decreased (Fig 3). However, these responses showed irregular patterns across concentration levels and only led to a significant contrast between the control and the different moxidectin concentrations in case of MGT and Z. Two-way ANOVA revealed that there were general differences between species and that in case of MGT and Z a species × treatment interaction existed (Table 3). This response pattern was corroborated when analysing for treatment effects on the species level (S2 Fig). Only in *P*. *lanceolata* MGT and Z (but not GP) differed between control and different moxidectin levels. The other two species showed no significant response. The weaker effects of moxidectin compared to Cydectin were corroborated when we tested the treatments with Cydectin vs. moxidectin with corresponding concentrations of the pharmaceutical drug moxidectin, i.e. 1 mg l^-1^ and 10 mg l^-1^. In both concentrations, mean GP was lower in case of seed exposure to Cydectin rather than moxidectin. However, this effect was only significant at the higher moxidectin concentration (1 mg l^-1^: 29.1 ± 3.2 vs. 33.6 ± 4.3; 10 mg l^-1^: 12.0 ± 1.1 vs. 33.1 ± 3.9; Cydectin vs. moxidectin; mean ± SE).

**Table 3:** Results of the ANOVA for the germination experiment with four different concentrations of moxidectin (0.01/0.1/1/10 mg l^-1^).

		GP			MGT			Z		
	df	MS	*F*	*P*	MS	*F*	*P*	MS	*F*	*P*
Intercept	1	53.55	4620.2	< 0.0001	16127.1	3632.9	< 0.0001	4.37	1113.78	< 0.0001
Species [S]	2	3.38	291.4	< 0.0001	141.9	31.97	< 0.0001	0.07	17.83	< 0.0001
Treatment [T]	4	0.017	1.5	0.22	8.1	1.83	0.13	0.02	4.15	0.003
S × T	8	0.018	1,6	0.13	13.6	3.05	0.003	0.01	2.53	0.014
Residuals	133	0.012			4.4			0.004		

Effects of species, treatment and the interaction of these factors on germination percentage (GP), mean germination time (MGT) and synchrony of germination (Z) are shown. Further abbreviations see Table 1.

## Discussion

The results presented are the first proof that anthelmintics can have adverse effects on seed germination. We found that the commonly used anthelmintic Cydectin (0.1% moxidectin) can reduce seedling number by almost two thirds and lead to a 12-d delay of germination when applied in a 10 mg l^-1^ concentration. Already a 7-d delay of germination can lead to significantly reduced plant fitness in terms of biomass and flower production as has been shown for the arable weed species *Agrostemma githago* [37]. In contrast to our results, an authorisation study that tested the germination response of 12 herbaceous plant species to moxidectin did not find adverse effects [11]. However, in the cited study specific laboratory conditions were tested, i.e. moxidectin was applied to a seed-soil system. Since moxidectin tightly binds to soil substrate [11], this might have caused seeds to only have come into contact with low concentrations of moxidectin.

The results of our germination experiments suggest that moxidectin acts more strongly on seeds if administered in formulation (Cydectin) than if administered solely. There are various possible explanations for this phenomenon. A crucial point in ecotoxicological test setups conducted with substances of low water solubility is a realistic exposition of the test organisms to the test substance. It is possible, that the seeds in the germination experiment treated with pure moxidectin were not exposed to the full moxidectin concentrations. Moxidectin might not have been completely dissolved and it might have been partly adsorbed by solid surfaces. This suggests a possible underestimation of effect size. This idea is supported by the fact that we found clear responses, both in case of the Cydectin solutions and in the feeding experiment under realistic conditions. In addition, the excipients of Cydectin might improve exposure of the seeds to the active ingredient in a synergistic way or the excipients itself could show adverse effects on seed germination. This could be addressed by working with the blank formulation of Cydectin without moxidectin. Unfortunately, mixtures of blank excipients are not provided by the producers. This is a general problem of a critical and free environmental research on pharmaceuticals, biocides and pesticides. Whatever the explanation for the observed differences between the pure active ingredient and its formulation might be, since in agricultural practice the formulations are used and not the isolated active ingredients, we suggest that further research should prioritise testing the formulations according to the concentrations recommended by the producer.

In addition to the specific effects of Cydectin (moxidectin), the pH value of the anthelmintic solutions represents a possible confounding factor that may also inhibit seed germination and seedling development [38]. The measured pH values (pH meter pH522, WTW, Germany; glass electrode SenTix21, WTW) of the used anthelmintic solutions were quite similar (Cydectin 1 mg l^-1^/10 mg l^-1^: 6.2/6.4; moxidectin 0.01/0.1/1/10 mg l^-1^: 5.6/5.7/5.7/5.8). These values lay within a range where no significant effects on germination can be expected for many species [38]. Therefore, we do not consider pH a relevant factor here.

From a physiological point of view, the question arises whether chloride ion channels of plants are affected by moxidectin as is the case in invertebrates. Plant chloride channels probably play an important role in signal transduction and many plant ion-channel types (including chloride channels) show homology to animal genes [39]. However, there is still not much known about the molecular characterisation and physiological role of plant chloride channels and further research is required to elucidate interactions of macrocyclic lactones with chloride channels [2, 39–40].

The observed adverse effects of Cydectin (moxidectin) will depend also on the environmental conditions. There are many factors that potentially influence the effects of anthelmintics on seeds and varying responses can be expected for different grazing systems: seed characteristics (size, shape, hardness of seed coat, retention time in the gastrointestinal tract of an ungulate species), animal characteristics (type of digestive tract, feeding and defecation behavior), drug characteristics (formulation) and livestock management (dose, route, time point and frequency of anthelmintic administration; e.g. [41]). These factors are partly related to each other, e.g. seed retention time depends on various animal characteristics [42]. Highest concentrations of moxidectin and its residues in cattle and horse faeces have been determined a few days after application [9, 43]. Seeds eaten soon after anthelmintic treatment might thus be more vulnerable to effects impeding germination. In the case of our experiment, seeds and anthelmintic solution were administered to sheep within a short time period on the same day and we analysed faeces defecated one or two days after treatment. Thus, we assume that the maximum impact of Cydectin has been assessed by our feeding experiment.

These patterns are of practical relevance, since seed-dispersal limitation is a major constrain to grassland phytodiversity in the fragmented cultural landscape of Europe [21, 44]. Restoration projects aim at improving seed exchanges between isolated plant populations [45] that are threatened by genetic bottlenecks [46]. Besides techniques such as the transfer of seed-containing plant material or seeding (reviewed in [47]), the guidance of mobile livestock herds is suggested as a restoration measure improving an exchange of seeds over short and long distances [48]. The protection of the high capacity of domestic ungulate species in transferring seeds, particularly via faeces [49], becomes increasingly important with respect to ongoing land use and climate changes.

From our results we conclude that anthelmintics may impact plant regeneration not only indirectly (reduced breakdown of faeces) but also directly through toxic effects. Further studies are necessary to test a broad spectrum of anthelmintics (their active ingredients represent various modes of action; [4]) and plant as well as ungulate species. Especially those approaches are required that test anthelmintics under realistic rangeland conditions. Studies on arthropods suggest that moxidectin is of lesser ecotoxicological risk than other anthelmintics [9, 50]. This active ingredient rather persists in faecal pats and does not spread easily in the broader environment by wash-off; it strongly binds to soil particles and is sensitive to photodegradation [11]. Nonetheless, we found evidence for adverse effects of moxidectin on seed germination and study of other—potentially more harmful—active ingredients or formulations seems to be required. To prevent a loss in dispersal efficiency, we recommend that domestic ungulates should not be kept on grasslands that are of high nature conservation value soon after they have been treated with moxidectin or other macrocyclic lactones.

## Supporting Information

S1 Fig Effects (mean + SE) of treatments (C = control, C1 = 1 mg l^-1^ Cydectin, C2 = 10 mg l^-1^ Cydectin) on germination of *Plantago lanceolata* (upper row), *Galium verum* (middle row) and *Centaurea jacea* (bottom row). Left column: germination percentage (GP) [%], middle column: mean germination time (MGT) [days], right column: synchrony of germination (Z) [unitless]. Lines below the bars indicate significance as revealed by contrast analyses; broken lines indicate significant differences at *P* ≤ 0.05; the upper lines indicate significance between C vs. C1 and C2, and the lower lines indicate differences between C1 and C2.
(TIFF)

S2 Fig Effects (mean + SE) of treatments (C = control, M1 = 0.01 mg l^-1^ moxidectin, M2 = 0.1 mg l^-1^ moxidectin, M3 = 1 mg l^-1^ moxidectin, M4 = 10 mg l^-1^ moxidectin) on germination of *Plantago lanceolata* (upper row), *Galium verum* (middle row) and *Centaurea jacea* (bottom row). Left column: germination percentage [%], middle column: mean germination time [days]; right column: synchrony of germination [unitless]. Lines below the bars indicate significance as revealed by contrasts of C vs. M1, M2, M3 and M4; broken lines indicate significant differences at *P* ≤ 0.05. No significant differences (*P* ≤ 0.05) were detected among treatments M1 to M4.
(TIFF)

S1 File The primary data of the feeding experiment. (PDF)
(PDF)

S2 File The primary data of the germination experiment. (PDF)
(PDF)
