# Supplementary figures and images for: The Anthelmintic Ingredient Moxidectin Negatively Affects Seed Germination of Three Temperate Grassland Species
Source: PLoS One. 2016 Nov 15;11(11):e0166366. doi: 10.1371/journal.pone.0166366 (PMC5112930; doi:10.1371/journal.pone.0166366)

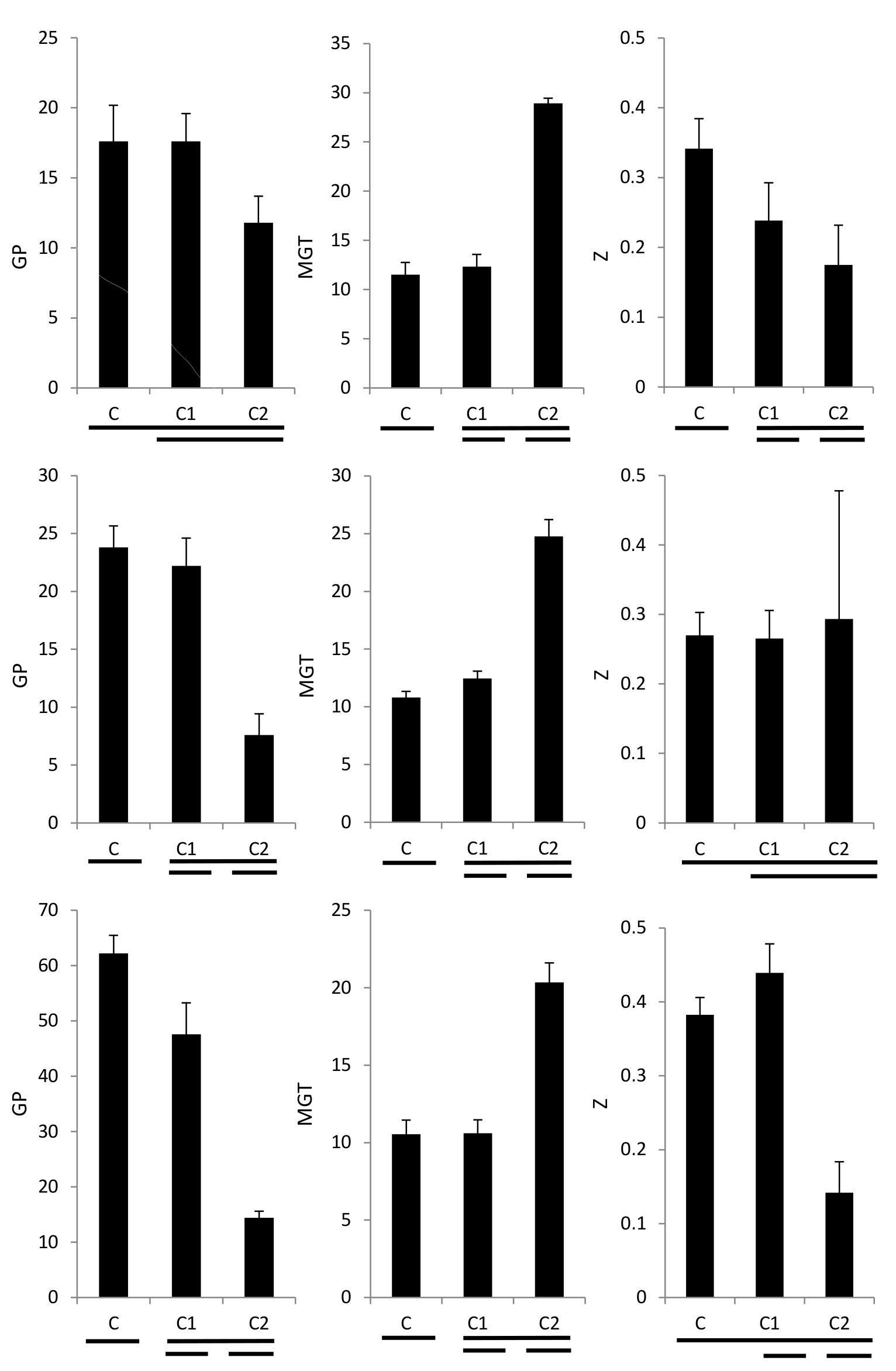

Supplement: S1 Fig — Left column: germination percentage (GP) [%], middle column: mean germination time (MGT) [days], right column: synchrony of germination (Z) [unitless]. Lines below the bars indicate significance as revealed by contrast analyses; broken lines indicate significant differences at P ≤ 0.05; the upper lines indicate significance between C vs. C1 and C2, and the lower lines indicate differences between C1 and C2. (TIFF) [file pone.0166366.s001.tiff]

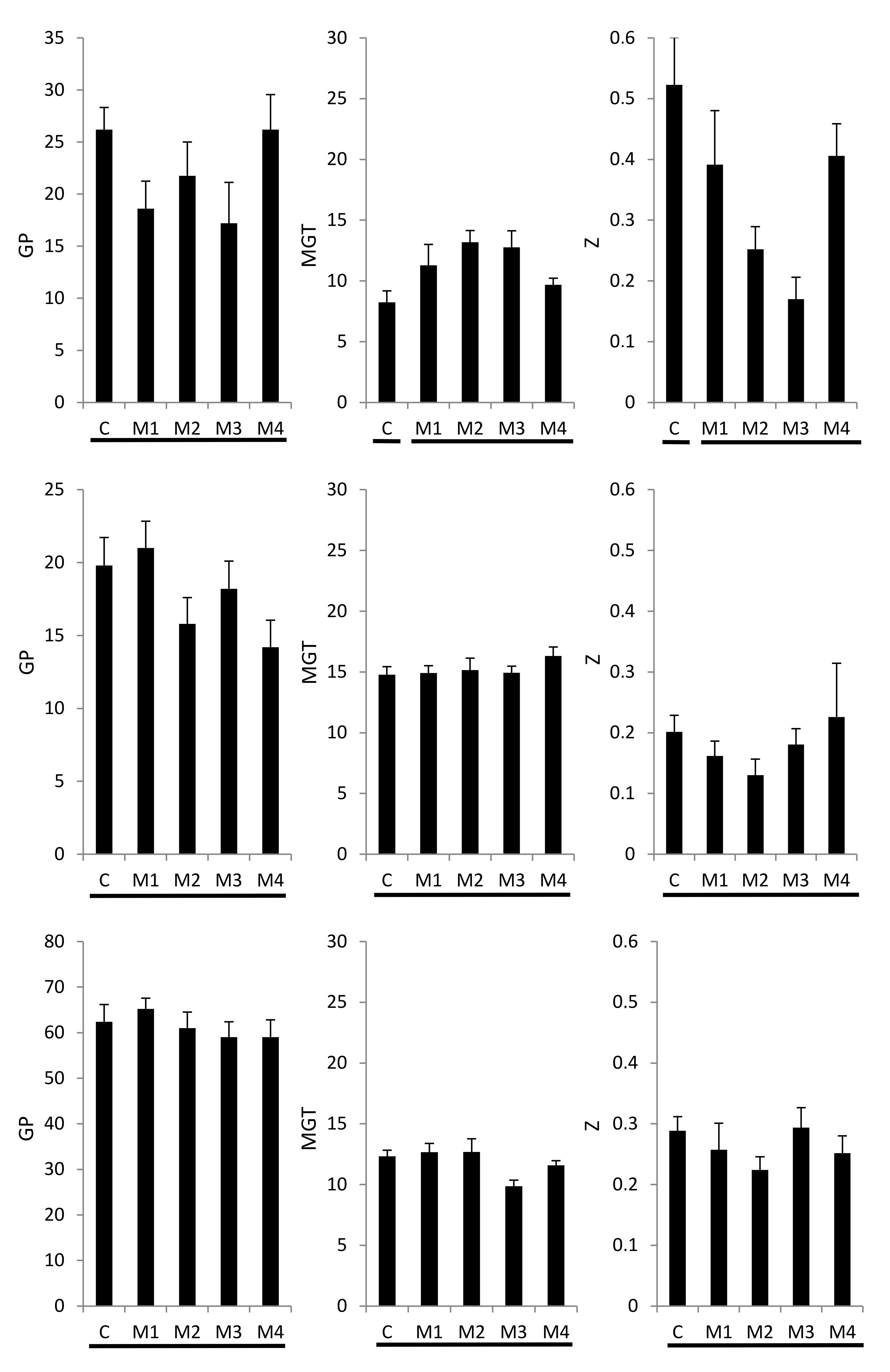

Supplement: S2 Fig — Left column: germination percentage [%], middle column: mean germination time [days]; right column: synchrony of germination [unitless]. Lines below the bars indicate significance as revealed by contrasts of C vs. M1, M2, M3 and M4; broken lines indicate significant differences at P ≤ 0.05. No significant differences (P ≤ 0.05) were detected among treatments M1 to M4. (TIFF) [file pone.0166366.s002.tiff]
