# Supplementary material for: The Anthelmintic Ingredient Moxidectin Negatively Affects Seed Germination of Three Temperate Grassland Species
Source: PLoS One. 2016 Nov 15;11(11):e0166366. doi: 10.1371/journal.pone.0166366 (PMC5112930; doi:10.1371/journal.pone.0166366)
Supplement: S1 File — (PDF) (PDF) [file pone.0166366.s003.pdf]

S1 File. The primary data of the feeding experiment.

Eichberg et al.

The Anthelmintic Ingredient Moxidectin Negatively Affects Seed Germination

Treatment	Day of faeces collection	Mean number of emerged seedlings per 100 g dry faeces
no Cydectin applied	1	33.1
no Cydectin applied	1	71.8
no Cydectin applied	1	54.4
no Cydectin applied	1	81.6
no Cydectin applied	1	22.6
no Cydectin applied	1	49.8
Cydectin applied	1	41.1
Cydectin applied	1	70.7
Cydectin applied	1	0
Cydectin applied	1	7.1
Cydectin applied	1	35.8
Cydectin applied	1	47.0
Cydectin applied	1	30.1
Cydectin applied	1	13.8
no Cydectin applied	2	3.0
no Cydectin applied	2	0
no Cydectin applied	2	3.3
no Cydectin applied	2	149.0
no Cydectin applied	2	0
no Cydectin applied	2	0
Cydectin applied	2	0
Cydectin applied	2	2.3
Cydectin applied	2	1.9
Cydectin applied	2	0
Cydectin applied	2	0
Cydectin applied	2	0
Cydectin applied	2	0
Cydectin applied	2	0
