# Supplementary material for: The Anthelmintic Ingredient Moxidectin Negatively Affects Seed Germination of Three Temperate Grassland Species
Source: PLoS One. 2016 Nov 15;11(11):e0166366. doi: 10.1371/journal.pone.0166366 (PMC5112930; doi:10.1371/journal.pone.0166366)
Supplement: S2 File — (PDF) (PDF) [file pone.0166366.s004.pdf]

S2 File. The primary data of the germination experiment.
Eichberg et al.
The Anthelmintic Ingredient Moxidectin Negatively Affects Seed Germination

		**Number of germinated seeds at day *x* after start of experiment**									
**Species**	**Treatment**	**4**	**8**	**11**	**15**	**18**	**22**	**25**	**29**	**32**	**36**
Centaurea jacea	blank control	0	20	6	3	2	3	2	0	0	1
Centaurea jacea	blank control	0	16	2	1	1	1	1	0	0	3
Centaurea jacea	blank control	0	18	2	1	2	2	1	4	0	1
Centaurea jacea	blank control	0	16	5	5	4	3	0	1	0	0
Centaurea jacea	blank control	0	24	2	2	2	2	1	1	0	2
Centaurea jacea	blank control	21	6	0	1	0	2	0	2	0	1
Centaurea jacea	blank control	14	4	0	1	0	1	0	0	0	0
Centaurea jacea	blank control	19	7	1	1	2	1	0	0	0	0
Centaurea jacea	blank control	8	17	1	0	2	2	4	0	0	0
Centaurea jacea	blank control	17	7	3	0	0	0	1	2	0	0
Centaurea jacea	solvent control	0	5	10	2	1	1	0	1	1	0
Centaurea jacea	solvent control	0	13	15	2	1	1	0	3	0	0
Centaurea jacea	solvent control	0	9	13	9	1	3	0	1	1	2
Centaurea jacea	solvent control	0	4	13	1	2	4	0	1	0	0
Centaurea jacea	solvent control	0	8	17	5	1	2	3	1	0	1
Centaurea jacea	solvent control	8	11	1	0	1	2	1	2	0	0
Centaurea jacea	solvent control	4	20	2	0	0	0	2	1	0	1
Centaurea jacea	solvent control	7	14	4	2	1	0	3	0	1	3
Centaurea jacea	solvent control	9	15	0	1	0	0	1	2	0	1
Centaurea jacea	solvent control	6	16	1	5	1	0	2	2	0	1
Centaurea jacea	C1	1	27	4	2	0	2	0	0	0	0
Centaurea jacea	C1	1	21	2	4	1	1	0	2	0	0
Centaurea jacea	C1	1	25	1	2	1	1	0	2	0	0
Centaurea jacea	C1	1	19	0	2	1	3	0	1	0	0
Centaurea jacea	C1	1	14	1	3	0	0	0	0	0	0
Centaurea jacea	C1	7	6	1	0	0	0	2	1	0	2
Centaurea jacea	C1	8	5	0	0	0	0	0	3	0	4
Centaurea jacea	C1	0	4	0	1	0	0	0	0	0	1
Centaurea jacea	C1	15	9	0	0	1	1	0	1	0	0
Centaurea jacea	C1	13	4	1	0	0	1	0	0	0	0
Centaurea jacea	C2	1	0	1	2	1	0	0	1	1	2
Centaurea jacea	C2	1	0	1	1	1	0	0	1	1	1
Centaurea jacea	C2	0	1	1	1	0	0	0	1	1	3
Centaurea jacea	C2	1	0	0	0	1	0	0	1	0	1
Centaurea jacea	C2	0	2	1	1	0	1	0	2	1	1
Centaurea jacea	C2	0	0	1	0	0	0	1	1	2	0
Centaurea jacea	C2	0	5	0	0	1	0	0	2	1	0
Centaurea jacea	C2	0	4	0	0	0	1	0	1	0	0
Centaurea jacea	C2	0	3	0	0	0	2	0	3	0	1
Centaurea jacea	C2	1	3	0	0	0	0	0	1	0	1
Centaurea jacea	M1	3	4	8	1	5	2	6	3	0	1
Centaurea jacea	M1	4	12	5	2	3	2	5	0	0	0
Centaurea jacea	M1	2	8	5	1	4	0	4	2	0	0
Centaurea jacea	M1	12	9	3	1	5	1	4	1	0	0
Centaurea jacea	M1	2	9	6	3	8	0	3	1	2	1
Centaurea jacea	M1	2	15	5	1	0	3	0	0	0	4
Centaurea jacea	M1	6	10	11	0	2	0	0	1	0	2
Centaurea jacea	M1	6	17	4	3	1	2	0	0	2	0
Centaurea jacea	M1	1	17	4	3	2	1	0	0	0	0
Centaurea jacea	M1	2	29	3	0	0	1	1	1	0	1
Centaurea jacea	M2	0	8	12	3	1	5	3	1	0	0

Centaurea jacea	M2	0	2	9	5	2	2	0	1	1	0
Centaurea jacea	M2	0	8	9	7	2	6	1	4	1	1
Centaurea jacea	M2	1	4	6	3	1	10	1	3	1	1
Centaurea jacea	M2	2	12	8	4	3	3	0	0	1	1
Centaurea jacea	M2	10	6	4	0	2	2	1	2	0	0
Centaurea jacea	M2	9	1	3	1	4	2	1	1	1	0
Centaurea jacea	M2	19	6	4	1	2	3	2	0	0	0
Centaurea jacea	M2	17	5	2	2	1	1	1	1	0	0
Centaurea jacea	M2	14	9	2	1	0	1	0	1	1	0
Centaurea jacea	M3	7	7	2	0	2	1	0	2	0	0
Centaurea jacea	M3	19	3	2	1	1	1	0	0	1	1
Centaurea jacea	M3	17	5	2	1	3	3	0	3	0	0
Centaurea jacea	M3	15	6	0	0	2	2	1	0	0	0
Centaurea jacea	M3	18	5	0	1	0	0	2	0	1	0
Centaurea jacea	M3	5	17	4	3	1	1	2	0	1	0
Centaurea jacea	M3	9	14	3	2	0	0	0	1	0	0
Centaurea jacea	M3	7	16	6	1	1	3	4	2	0	0
Centaurea jacea	M3	4	7	8	3	3	3	1	0	0	0
Centaurea jacea	M3	6	9	5	3	1	0	2	0	0	0
Centaurea jacea	M4	2	13	6	1	0	3	2	0	0	0
Centaurea jacea	M4	6	11	4	1	2	3	3	1	0	0
Centaurea jacea	M4	8	12	2	0	2	2	1	2	0	4
Centaurea jacea	M4	5	15	7	0	2	1	1	2	0	1
Centaurea jacea	M4	3	5	4	1	2	1	0	1	0	0
Centaurea jacea	M4	8	16	0	0	2	0	1	2	1	0
Centaurea jacea	M4	5	22	2	0	3	1	0	1	0	0
Centaurea jacea	M4	5	12	2	1	1	2	1	1	0	0
Centaurea jacea	M4	5	7	4	1	4	4	1	0	0	0
Centaurea jacea	M4	3	19	4	5	3	0	1	1	0	2
Galium verum	blank control	1	6	3	1	1	0	0	0	0	0
Galium verum	blank control	2	6	4	0	0	1	0	1	0	0
Galium verum	blank control	3	7	1	0	1	0	0	1	0	0
Galium verum	blank control	1	6	0	1	0	0	0	0	0	0
Galium verum	blank control	0	6	5	2	2	0	1	1	0	0
Galium verum	blank control	0	4	3	1	0	0	0	0	1	0
Galium verum	blank control	0	4	5	1	1	0	0	0	0	0
Galium verum	blank control	2	6	3	0	2	0	0	0	0	0
Galium verum	blank control	2	3	2	0	0	0	1	0	0	0
Galium verum	blank control	1	5	2	3	1	0	0	2	0	0
Galium verum	solvent control	0	1	3	2	0	0	0	1	0	0
Galium verum	solvent control	0	2	7	0	2	0	0	0	0	0
Galium verum	solvent control	0	3	6	2	1	0	0	1	0	0
Galium verum	solvent control	0	1	3	1	0	0	0	2	0	0
Galium verum	solvent control	0	2	2	2	1	0	0	0	0	0
Galium verum	solvent control	0	5	2	4	1	1	1	1	0	0
Galium verum	solvent control	0	0	3	0	1	1	0	1	0	0
Galium verum	solvent control	0	2	0	3	1	2	1	1	0	0
Galium verum	solvent control	0	2	5	3	0	1	0	0	0	1
Galium verum	solvent control	1	2	0	1	2	4	1	0	0	0
Galium verum	C1	0	4	3	1	1	0	0	2	0	0
Galium verum	C1	0	3	1	1	1	0	0	1	0	0
Galium verum	C1	0	5	5	1	0	1	0	2	0	0
Galium verum	C1	0	6	2	0	1	0	0	0	0	0
Galium verum	C1	0	3	2	0	0	0	0	1	0	0
Galium verum	C1	0	5	2	0	0	0	0	0	0	0
Galium verum	C1	1	6	3	2	2	0	0	1	0	0
Galium verum	C1	0	4	3	1	0	0	1	1	0	1
Galium verum	C1	0	8	2	1	1	0	1	1	0	0

Galium verum	C1	1	5	6	2	1	1	1	0	0	0
Galium verum	C2	0	0	1	0	0	0	0	1	0	0
Galium verum	C2	0	0	0	0	0	0	0	0	0	0
Galium verum	C2	0	1	0	0	0	0	0	0	0	0
Galium verum	C2	0	0	0	0	0	0	0	0	0	0
Galium verum	C2	0	0	0	0	0	0	0	0	1	0
Galium verum	C2	0	0	1	0	0	0	0	1	0	1
Galium verum	C2	0	0	0	0	0	0	0	2	0	0
Galium verum	C2	0	0	0	0	0	0	0	0	0	0
Galium verum	C2	0	0	0	0	2	1	0	3	0	0
Galium verum	C2	0	0	0	0	0	2	2	2	0	0
Galium verum	M1	0	2	4	2	1	0	2	2	0	0
Galium verum	M1	0	4	4	1	4	1	1	0	0	0
Galium verum	M1	0	1	2	2	1	1	0	0	0	0
Galium verum	M1	0	2	2	1	2	1	1	1	0	0
Galium verum	M1	0	1	2	2	5	1	0	2	0	0
Galium verum	M1	0	1	3	3	1	0	1	0	0	1
Galium verum	M1	0	4	3	1	0	0	0	1	0	0
Galium verum	M1	0	2	1	2	1	0	0	0	0	0
Galium verum	M1	0	3	3	2	3	2	0	0	0	0
Galium verum	M1	0	0	2	5	2	0	0	0	0	0
Galium verum	M2	0	1	1	2	1	0	0	1	0	0
Galium verum	M2	0	2	3	1	2	0	1	0	0	0
Galium verum	M2	1	5	3	1	0	1	0	1	0	0
Galium verum	M2	1	2	1	2	1	0	1	1	0	0
Galium verum	M2	0	1	1	2	2	2	0	2	0	0
Galium verum	M2	0	2	2	4	0	0	0	1	0	0
Galium verum	M2	0	0	2	0	2	0	0	0	1	0
Galium verum	M2	0	0	1	1	0	0	0	0	0	0
Galium verum	M2	0	1	1	3	0	1	0	1	1	1
Galium verum	M2	0	4	2	1	1	0	0	0	0	0
Galium verum	M3	0	0	4	3	2	0	0	1	0	0
Galium verum	M3	0	1	5	2	2	1	0	1	0	0
Galium verum	M3	0	3	1	1	0	0	1	0	0	0
Galium verum	M3	0	3	4	2	3	0	0	0	0	0
Galium verum	M3	0	1	1	0	2	1	1	0	0	0
Galium verum	M3	0	2	7	1	0	2	1	0	0	0
Galium verum	M3	0	0	2	1	1	1	0	1	0	0
Galium verum	M3	0	0	4	2	2	0	0	0	0	0
Galium verum	M3	1	1	3	2	2	0	2	1	0	0
Galium verum	M3	0	0	3	1	1	0	0	1	0	0
Galium verum	M4	0	0	1	2	0	2	0	0	0	0
Galium verum	M4	0	0	3	1	1	1	0	0	0	0
Galium verum	M4	0	0	2	1	1	0	1	0	0	0
Galium verum	M4	0	0	3	2	2	1	1	1	0	0
Galium verum	M4	0	0	2	3	0	0	2	2	0	0
Galium verum	M4	0	2	1	1	1	0	1	1	1	1
Galium verum	M4	0	1	2	1	1	0	0	0	1	0
Galium verum	M4	0	3	2	1	0	0	1	0	0	0
Galium verum	M4	0	2	5	2	1	0	0	0	2	0
Galium verum	M4	0	0	0	2	0	0	0	0	0	0
Plantago lanceolata	blank control	3	1	0	0	0	0	1	0	0	0
Plantago lanceolata	blank control	5	1	0	0	0	0	0	0	1	0
Plantago lanceolata	blank control	6	0	0	1	1	1	0	2	3	1
Plantago lanceolata	blank control	0	2	0	0	0	2	0	0	0	0
Plantago lanceolata	blank control	10	1	0	0	0	1	0	1	0	0
Plantago lanceolata	blank control	1	2	0	0	0	0	0	0	1	0
Plantago lanceolata	blank control	4	4	1	0	0	1	0	0	0	0

Plantago lanceolata	blank control	1	6	0	0	0	1	0	1	2	1
Plantago lanceolata	blank control	3	8	0	0	0	1	0	0	0	0
Plantago lanceolata	blank control	0	4	1	0	0	1	0	0	0	0
Plantago lanceolata	solvent control	0	9	1	1	3	1	0	1	0	1
Plantago lanceolata	solvent control	2	7	0	1	1	0	0	1	0	0
Plantago lanceolata	solvent control	3	3	2	0	1	0	0	1	0	0
Plantago lanceolata	solvent control	1	11	0	0	2	0	0	0	0	0
Plantago lanceolata	solvent control	2	10	2	0	1	0	0	0	0	0
Plantago lanceolata	solvent control	7	3	1	1	0	0	1	0	0	0
Plantago lanceolata	solvent control	7	1	0	0	0	0	0	0	0	0
Plantago lanceolata	solvent control	8	0	0	1	0	0	0	0	0	0
Plantago lanceolata	solvent control	15	1	0	1	0	0	0	0	1	0
Plantago lanceolata	solvent control	14	0	0	0	0	0	0	1	0	0
Plantago lanceolata	C1	1	0	0	0	1	1	0	1	0	0
Plantago lanceolata	C1	3	2	1	2	0	0	0	1	0	1
Plantago lanceolata	C1	3	1	0	0	1	0	1	0	1	0
Plantago lanceolata	C1	3	1	0	0	1	0	1	0	0	0
Plantago lanceolata	C1	9	1	0	1	0	0	3	1	0	0
Plantago lanceolata	C1	3	0	0	3	1	0	2	0	3	0
Plantago lanceolata	C1	7	0	0	2	0	0	0	0	0	0
Plantago lanceolata	C1	4	1	0	2	0	0	0	1	0	0
Plantago lanceolata	C1	3	0	0	1	2	0	0	0	1	0
Plantago lanceolata	C1	6	1	0	2	1	0	0	0	0	0
Plantago lanceolata	C2	0	0	0	0	0	1	0	1	0	0
Plantago lanceolata	C2	0	0	0	0	0	0	1	5	2	0
Plantago lanceolata	C2	0	0	0	0	0	0	0	6	2	1
Plantago lanceolata	C2	0	0	0	0	1	0	0	3	1	2
Plantago lanceolata	C2	0	0	0	0	0	0	1	1	2	1
Plantago lanceolata	C2	0	0	0	0	0	2	1	1	1	1
Plantago lanceolata	C2	0	0	1	0	0	0	1	6	0	1
Plantago lanceolata	C2	0	0	0	0	0	1	2	3	2	1
Plantago lanceolata	C2	0	0	0	0	0	1	0	0	0	1
Plantago lanceolata	C2	0	0	0	0	0	0	1	0	0	1
Plantago lanceolata	M1	0	1	0	0	1	0	0	1	0	1
Plantago lanceolata	M1	1	7	2	0	0	0	0	2	1	0
Plantago lanceolata	M1	3	9	2	1	0	0	0	0	0	0
Plantago lanceolata	M1	0	2	0	0	0	0	0	0	0	0
Plantago lanceolata	M1	0	4	0	0	1	0	2	1	0	0
Plantago lanceolata	M1	3	4	0	0	0	0	0	3	0	0
Plantago lanceolata	M1	4	2	0	1	0	0	0	1	0	1
Plantago lanceolata	M1	5	6	1	0	0	0	0	1	0	0
Plantago lanceolata	M1	5	2	0	0	0	0	0	0	0	0
Plantago lanceolata	M1	10	0	1	0	1	0	0	0	0	0
Plantago lanceolata	M2	0	0	0	0	0	1	0	0	0	0
Plantago lanceolata	M2	0	5	0	0	0	0	0	2	1	0
Plantago lanceolata	M2	0	2	2	0	0	0	0	0	0	0
Plantago lanceolata	M2	1	3	0	0	0	1	0	0	0	1
Plantago lanceolata	M2	0	1	0	0	0	0	0	0	0	0
Plantago lanceolata	M2	4	2	3	0	0	2	0	1	0	3
Plantago lanceolata	M2	3	2	1	2	1	0	1	0	1	0
Plantago lanceolata	M2	8	1	0	2	0	1	1	1	1	1
Plantago lanceolata	M2	2	9	1	1	0	0	0	0	0	3
Plantago lanceolata	M2	5	1	4	0	0	0	0	1	0	0
Plantago lanceolata	M3	4	2	0	0	1	0	0	0	0	0
Plantago lanceolata	M3	2	1	0	2	0	0	0	0	0	0
Plantago lanceolata	M3	2	1	1	0	0	1	0	0	0	0
Plantago lanceolata	M3	1	0	0	1	0	1	0	3	0	0
Plantago lanceolata	M3	0	0	1	1	0	0	0	0	0	0

Plantago lanceolata	M3	1	3	1	0	0	0	0	0	0	3
Plantago lanceolata	M3	0	1	0	0	1	0	0	0	0	0
Plantago lanceolata	M3	6	6	0	0	0	4	0	0	0	0
Plantago lanceolata	M3	3	4	2	2	0	1	0	2	0	1
Plantago lanceolata	M3	5	8	0	0	2	4	1	0	0	0
Plantago lanceolata	M4	6	3	0	1	0	0	3	0	0	1
Plantago lanceolata	M4	2	1	0	1	0	0	0	0	0	0
Plantago lanceolata	M4	3	7	0	2	0	0	0	0	0	0
Plantago lanceolata	M4	6	7	0	1	1	0	0	0	0	0
Plantago lanceolata	M4	2	11	0	0	0	0	0	0	1	0
Plantago lanceolata	M4	7	9	1	0	1	0	1	1	1	0
Plantago lanceolata	M4	1	7	0	1	0	0	0	0	0	1
Plantago lanceolata	M4	3	11	0	0	1	0	1	0	0	0
Plantago lanceolata	M4	1	16	0	1	0	0	0	0	1	0
Plantago lanceolata	M4	0	4	0	1	0	0	1	0	0	0
